# Supplementary material for: Metallic micronutrients are associated with the structure and function of the soil microbiome
Source: Nat Commun. 2023 Dec 20;14:8456. doi: 10.1038/s41467-023-44182-2 (PMC10730613; doi:10.1038/s41467-023-44182-2)
Supplement: Supplementary file 1 — Supplementary Information [file 41467_2023_44182_MOESM1_ESM.pdf]

Supporting Information

Fig. S1. The distribution of 180 soil sites including 22 facility, 20 paddy, 38 forest, 42 upland, 31 grassland, 14 urban and 13 wetland soil samples collected across China. The base map used in Figure S1 was applied without endorsement using data from the Aliyun (http://datav.aliyun.com/portal/school/atlas/area_selector).

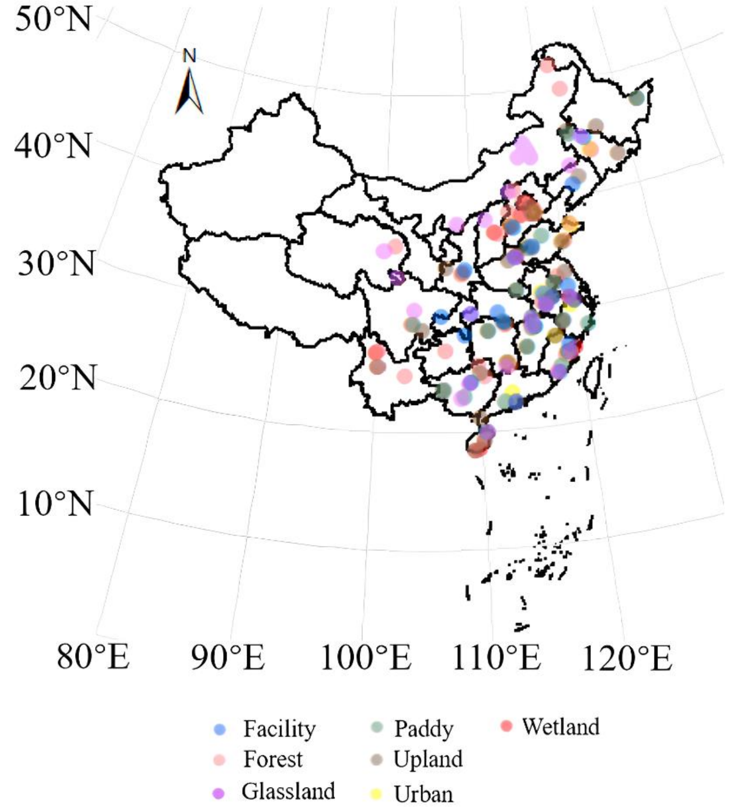

Fig. S2. The range of soil pH (a), total C (b), total N (c), total P (d) concentrations and macronutrient ratios (e) across 180 soil samples.

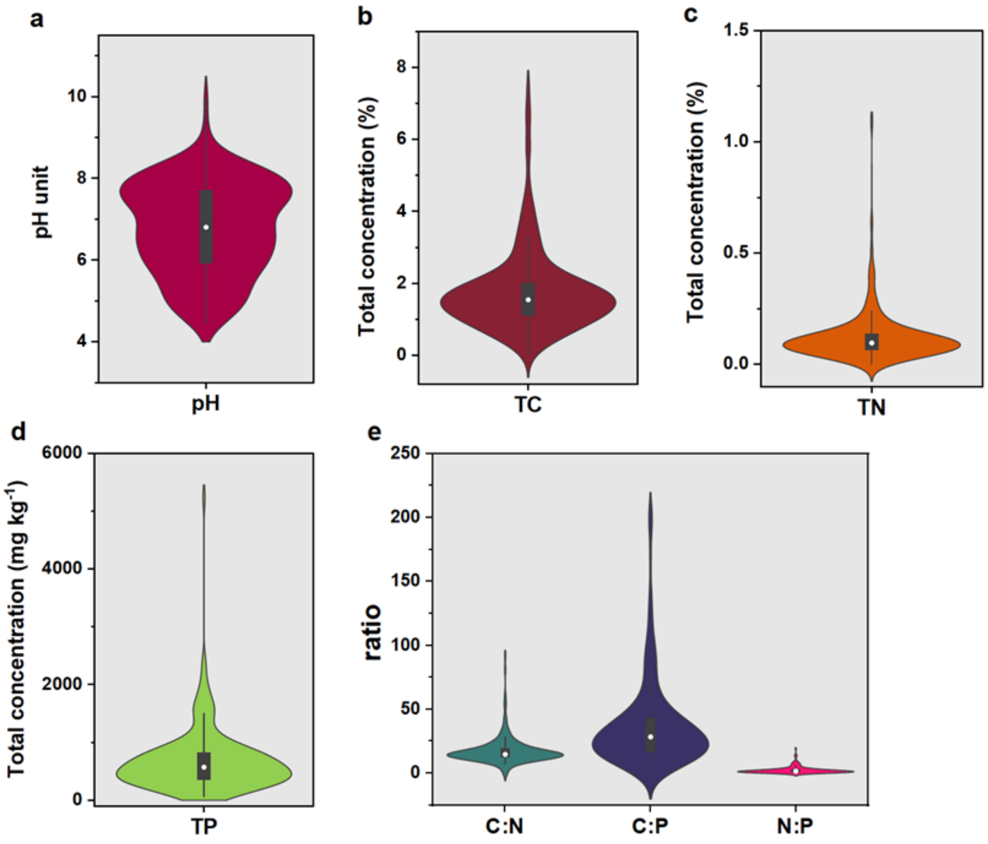

Fig. S3. The range of total (a and b) and available (c and d) micronutrient concentrations across 180 soil samples.

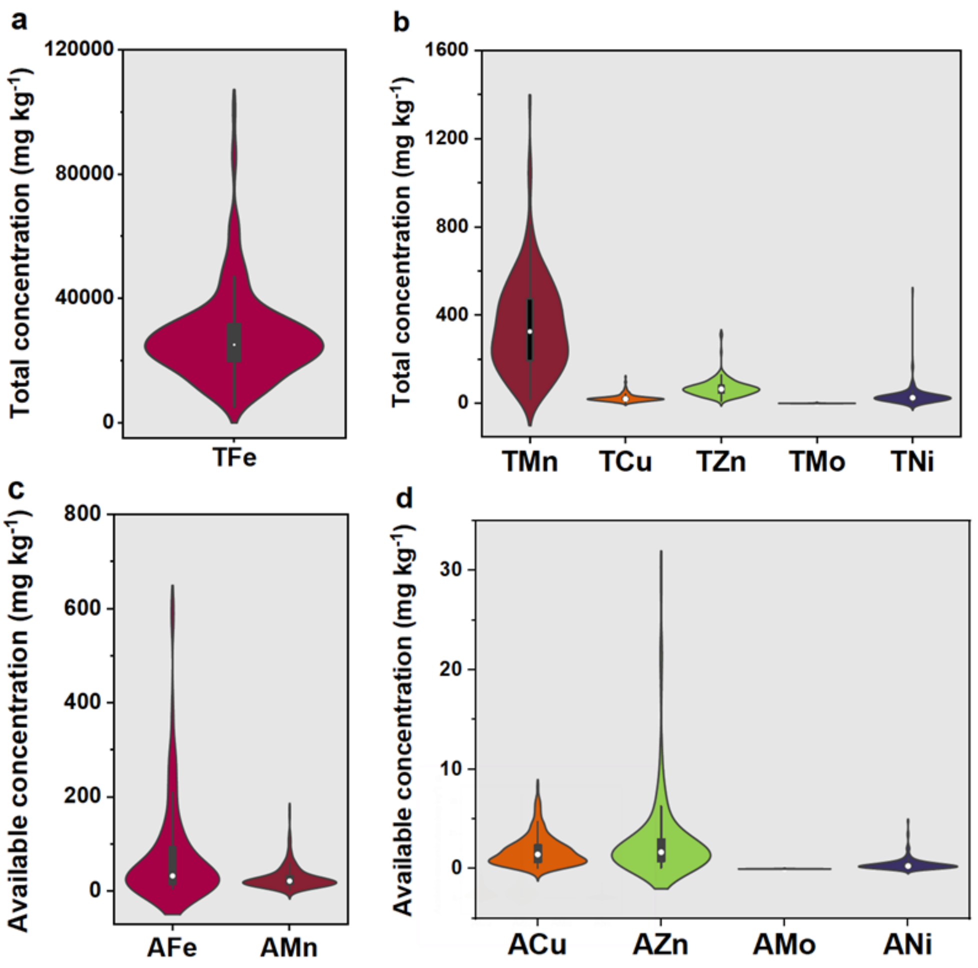

Fig. S4. Effects of total and available micronutrients (Fe, Mn, Cu, Zn, Mo, Ni) on soil microbial abundances. The relationships between micronutrients and bacterial abundance (a and b), and between fungal abundance (c and d) were conducted by the partial correlation. The total PC1 and total PC2 were obtained by using the principal component analysis of the total micronutrients, and the available PC1 and available PC2 were obtained using available micronutrients. The total PC1, total PC2, available PC1 and available PC2 represented the integrated effects of Fe, Mn, Cu, Zn, Mo and Ni. The solid dots represent the positive correlation coefficients and the empty dots represent the negative correlation coefficients in radar plots.

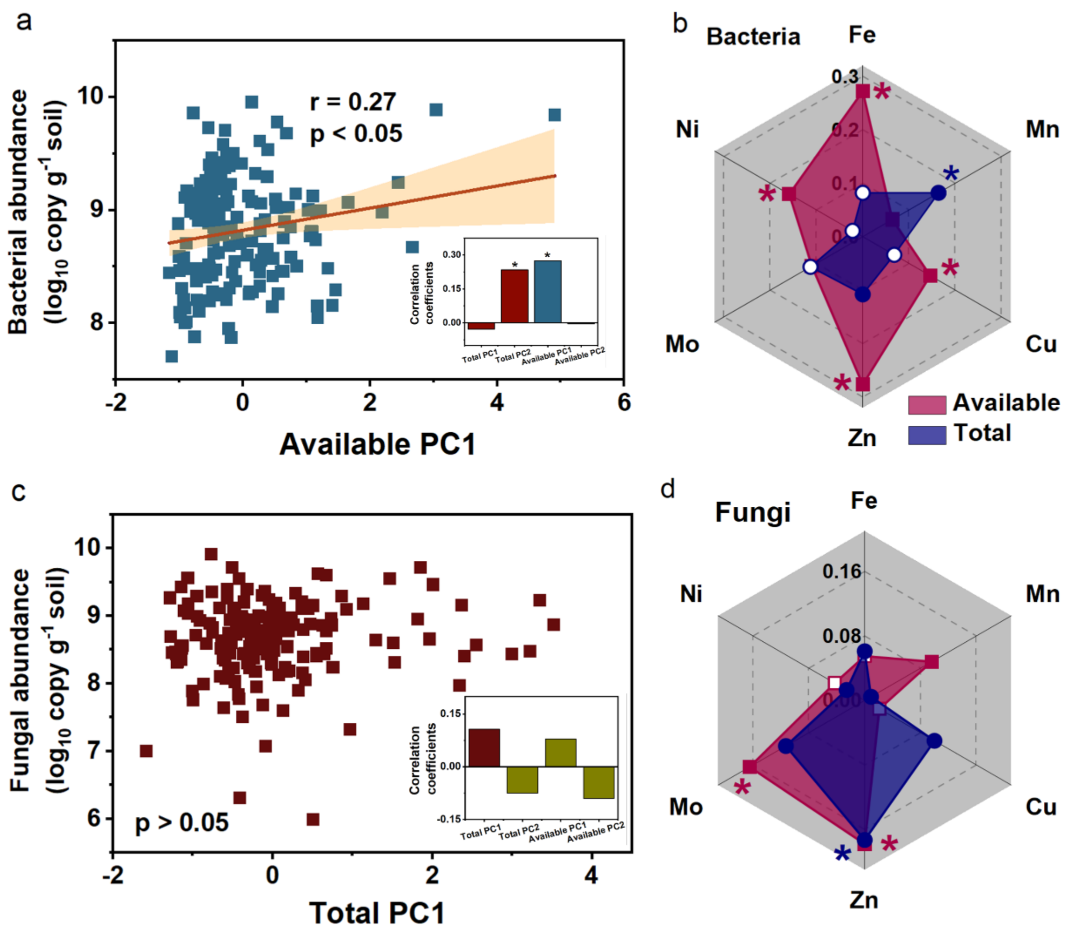

Fig. S5. Partial correlation for the relationship between soil pH, macronutrients and bacterial (a) and fungal (b) abundances. The solid dots represent the positive correlation coefficients and the empty dots represent the negative correlation coefficients in radar plots.

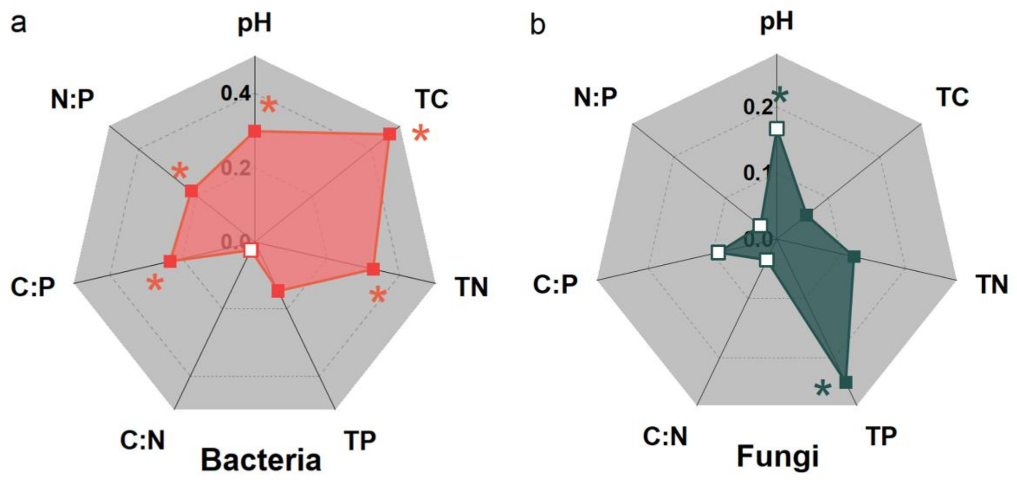

Fig. S6. The effects of available micronutrients (Fe, Mn, Cu, Zn, Mo, Ni) on the abundances of bacterial and fungal taxa at genus level. The identified genera had the relative abundances that were significantly higher or lower in relative low micronutrients as compared with high micronutrient group. The low group included the 60 samples with relative lower micronutrient concentrations in the total of 180 samples and the high group included the 60 samples with relative higher micronutrient concentrations in the 180 samples. The blue and red dots represent the identified genera whose abundance decreased and increased as available micronutrient concentrations increased respectively.

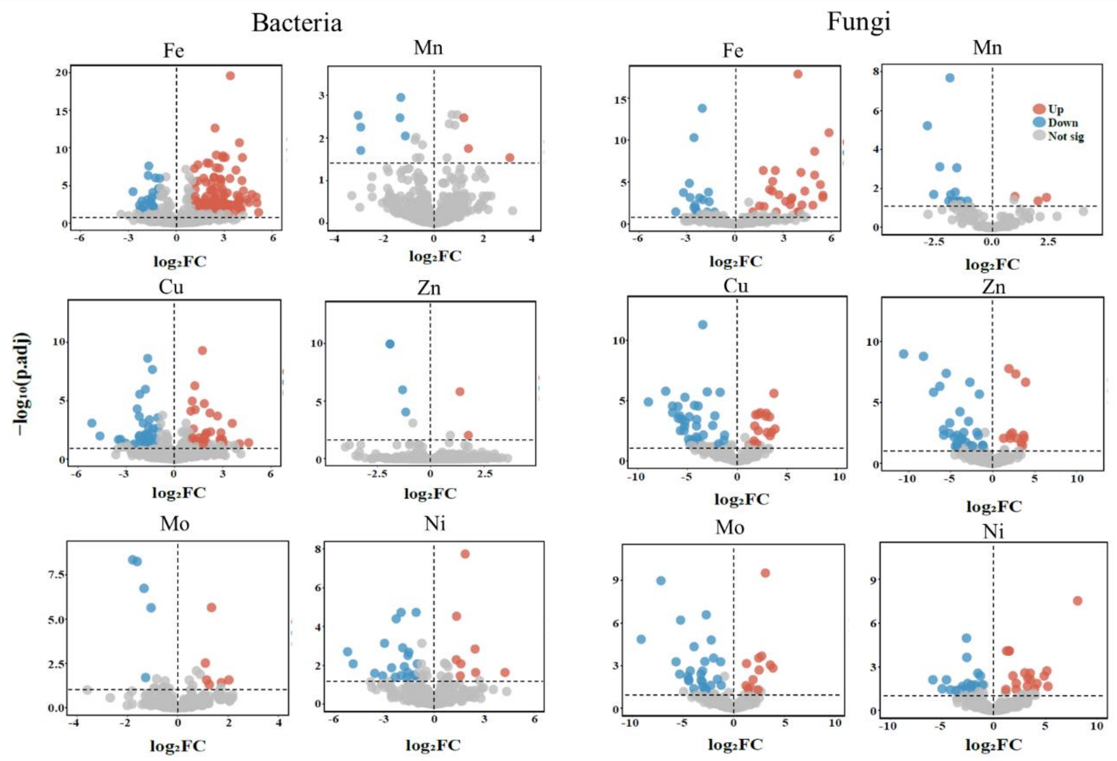

Fig. S7. The effects of total micronutrients (Fe, Mn, Cu, Zn, Mo, Ni) on the abundances of bacterial and fungal taxa at genus level. The identified genera had the relative abundances that were significantly higher or lower in relative low micronutrients as compared with high micronutrient group. The low group included the 60 samples with relative lower micronutrient concentrations in the total of 180 samples and the high group included the 60 samples with relative higher micronutrient concentrations in the 180 samples. The blue and red dots represent the identified genera whose abundance decreased and increased as total micronutrient concentrations increased respectively.

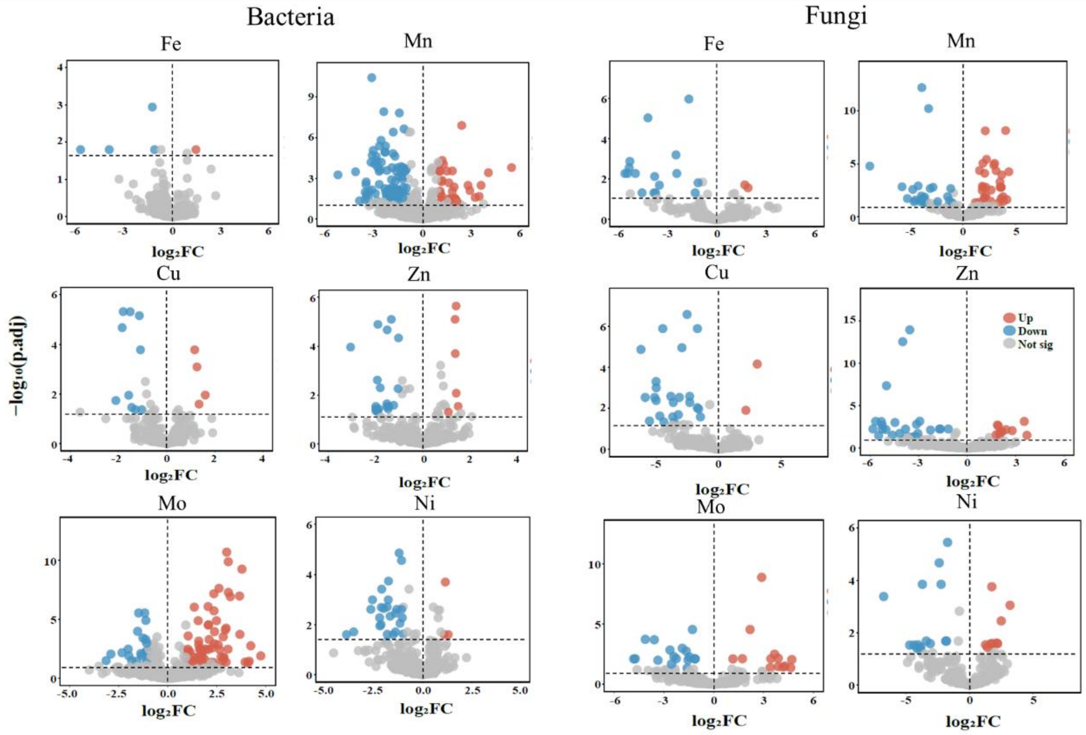

Fig. S8. The differences in soil bacterial (a and b) and fungal (c and d) communities as revealed by community dissimilarity between different climate zones and land uses, performed by two-way ANOVA.

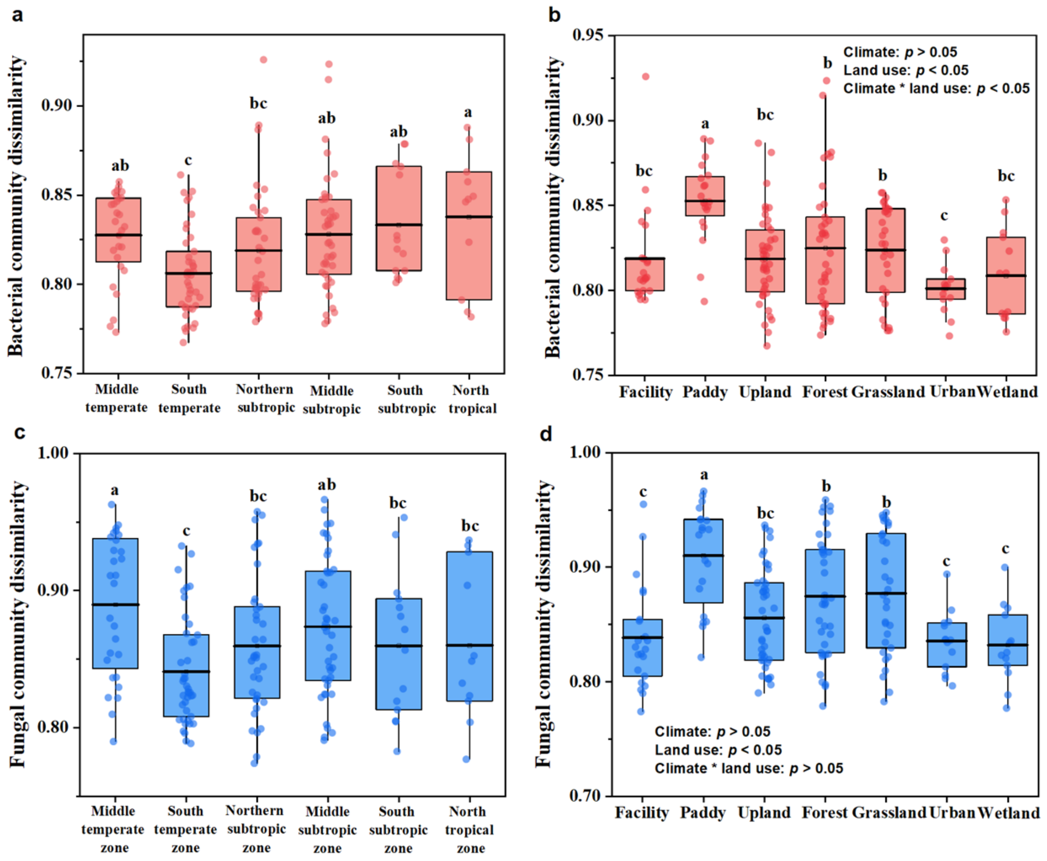

Fig. S9. Partial correlation coefficients (avoiding the pH effects) for the relationship between soil macronutrients and the alpha, beta diversity, the abundance of dominant phyla and network connectivity for bacteria (a) and fungi (b).

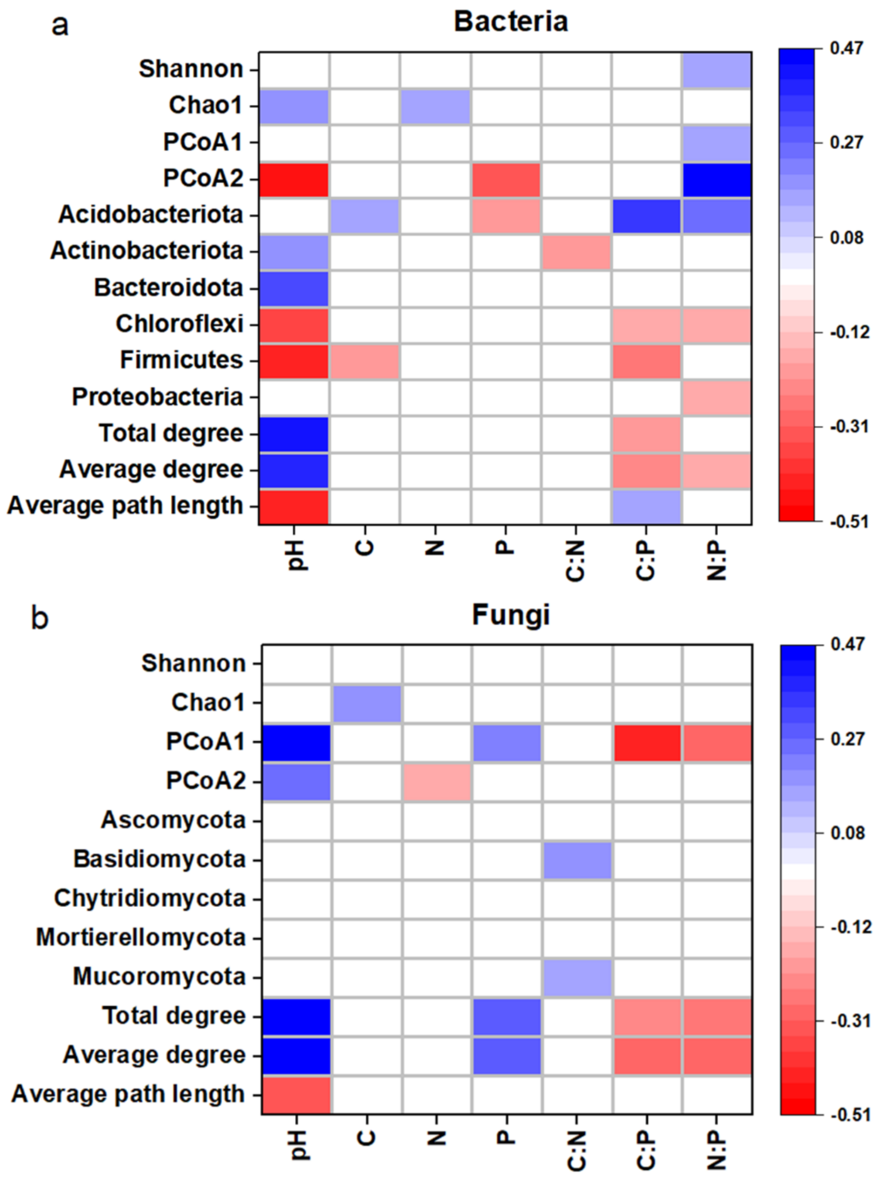

Fig. S10. Score coefficients of principal component 1 (PC1) and principal component 2 (PC2) for the total (a) and available (b) micronutrients across 180 soil samples, shown by the principal components analysis.

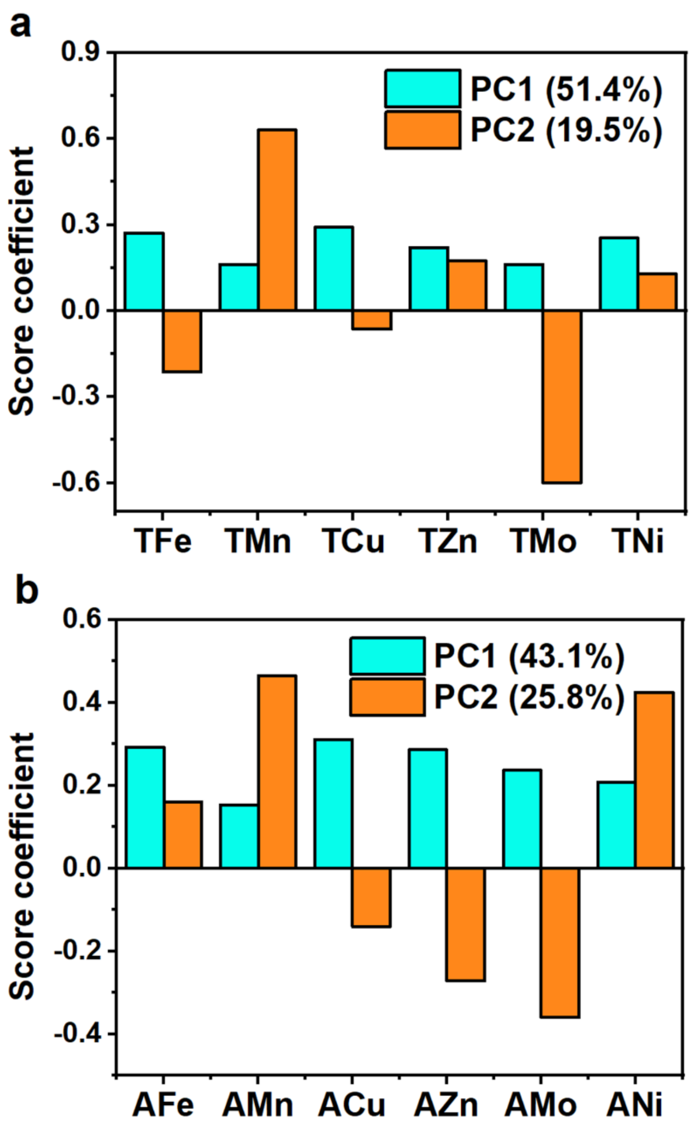

Fig. S11. The original paths of SEM hypothesizing how environmental factors contribute to ecosystem production.

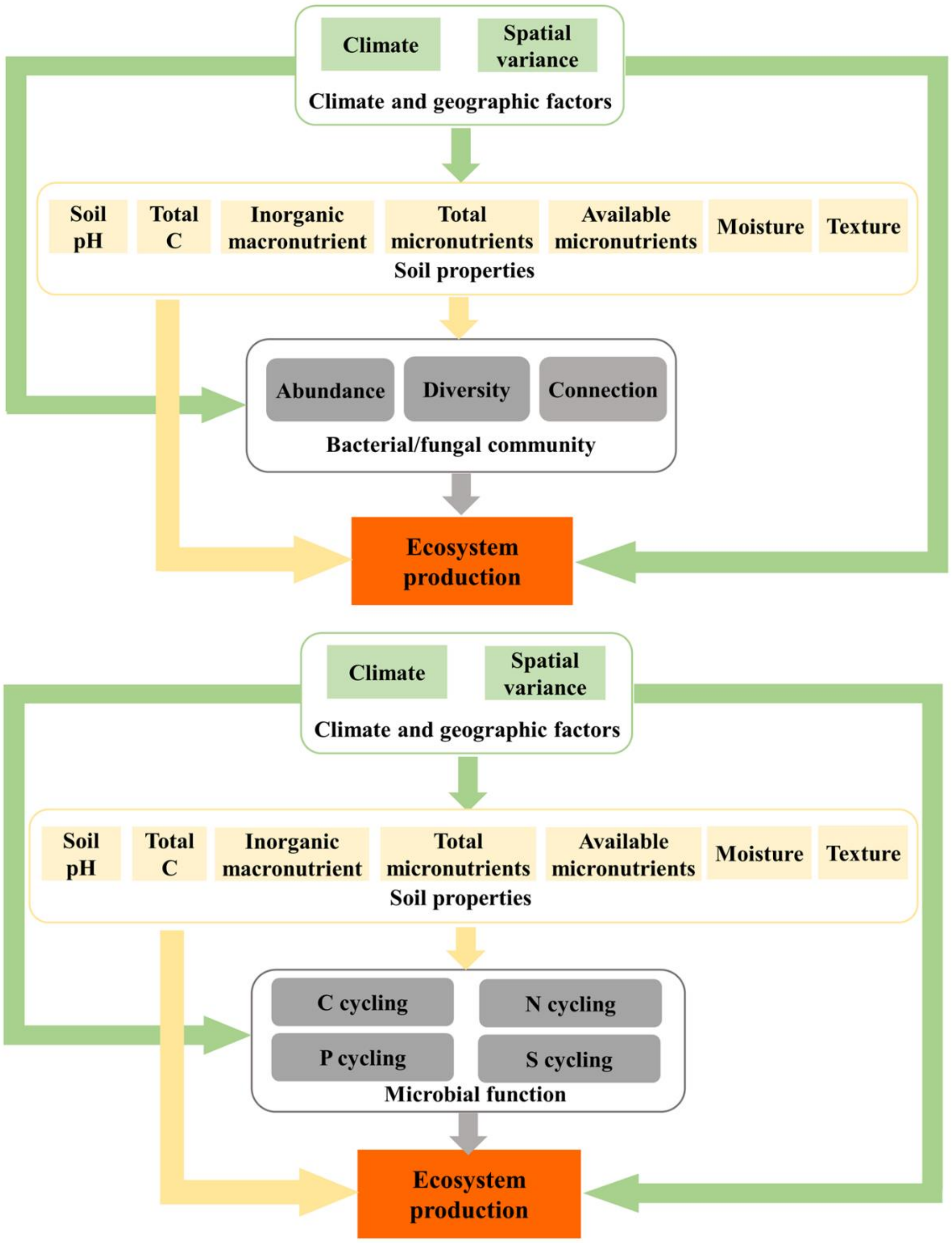

Table S1. The correlation coefficients of the correlations between the abundances of identified genera and micronutrient available concentrations with and without data normalization. The “*” represents that the correlation is significant at adjusted $p < 0.05$ (two-sided).

Micronutrient	Kingdom	Genera	No normalization	Normalization (Log10 transformation)
			Correlation coefficient (r)	Correlation coefficient (r)
	Bacteria	*0319-7L14*	-0.26*	-0.43*
		Pir4_lineage	-0.30*	-0.46*
		RBG-13-54-9	0.33*	0.33*
		SHA-26	0.40*	0.48*
Available Fe	Fungi	*Acremonium*	-0.17*	-0.23*
		Saitozyma	0.16*	0.22*
		Gibberella	-0.19*	-0.24*
		Scolecobasidium	0.32*	0.34*
	Bacteria	*0319-7L14*	-0.21*	-0.20*
		JG30-KF-AS9	0.22*	0.20*
		Pir4_lineage	-0.22*	-0.22*
		SHA-26	0.24*	0.25*
Available Mn	Fungi	*Arthrinium*	0.13	0.22
		Aspergillus	0.15*	0.19*
		Cephalotrichum	-0.31*	-0.30*
		Gibberella	-0.16*	-0.10
	Bacteria	*Bradyrhizobium*	-0.25*	-0.38*
		Rubrobacter	-0.21*	-0.29*
		SBR1031	0.23*	0.34*
		UTCFX1	0.22*	0.32*
Available Cu	Fungi	*Geminibasidium*	-0.20*	-0.19*
		Plectosphaerella	0.08	0.05
		Spizellomyces	0.19*	0.22*
		Trichoderma	-0.13	-0.17*
	Bacteria	*Actinomadura*	0.08	0.17*
		Candidatus_Udaeobacter	-0.16*	-0.23*
		Rubrobacter	-0.17*	-0.44*
		Streptomyces	0.29*	0.32*
Available Zn	Fungi	*Botryotrichum*	0.35*	0.32*
		Humicola	0.40*	0.32*
		Leohumicola	-0.18*	-0.18*
		Trichoderma	-0.17*	-0.18*

	Bacteria	*Candidatus_Udaeobacter*	-0.15*	-0.20*
		Rubrobacter	-0.18*	-0.22*
		Saccharopolyspora	0.17*	0.23*
		Streptomyces	0.28*	0.30*
Available Mo	Fungi	*Botryotrichum*	0.16*	0.22*
		Geminibasidium	-0.08	-0.11
		Kernia	0.19*	0.20*
		Trichoderma	-0.17*	-0.18*
	Bacteria	*Candidatus_Udaeobacter*	0.20*	0.22*
		Nocardioides	-0.20*	-0.26*
		Ralstonia	-0.07	-0.18*
		Anaerolinea	0.43*	0.28*
Available Ni	Fungi	*Cephalotrichum*	-0.17*	-0.16
		Fusarium	0.19*	0.35*
		Laburnicola	0.32*	0.16*
		Saitozyma	-0.09	-0.08

Table S2. The correlation coefficients of the correlations between the abundances of identified genera and micronutrient total concentrations with and without data normalization. The “*” represents that the correlation is significant at adjusted $p < 0.05$ (two-sided).

Micronutrient	Kingdom	Genera	No normalization	Normalization (Log10 transformation)
			Correlation coefficient (r)	Correlation coefficient (r)
	Bacteria	*Ralstonia*	0.24*	0.19*
		Rubrobacter	-0.25*	-0.36*
		Ignavibacterium	-0.38*	-0.44*
Total Fe	Fungi	*Cladorrhinum*	0.02	0.06
		Heydenia	-0.15*	-0.19*
		Plectosphaerella	-0.01	0.04
		Pleurophragmium	-0.24*	-0.34*
	Bacteria	*FCPS473*	-0.29*	-0.41*
		JG30-KF-AS9	-0.23*	-0.22*
		Pir4_lineage	0.25*	0.30*
		Promicromonospora	0.17*	0.19*
Total Mn	Fungi	*Gibberella*	0.17*	0.17*
		Pithoascus	0.16*	0.15*
		Saitozyma	-0.31*	-0.48*
		Trichoderma	-0.19*	-0.20*
	Bacteria	*Candidatus_Udaeobacter*	-0.20*	-0.21*
		Rubrobacter	-0.20*	-0.34*
		Saccharopolyspora	0.20*	0.27*
		UTCFX1	0.16*	0.21*
Total Cu	Fungi	*Cladorrhinum*	0.04	0.08
		Heydenia	-0.16*	-0.16*
		Trichobolus	0.05	0.05
		Trichoderma	-0.15*	-0.16*
	Bacteria	*Rubrobacter*	-0.24*	-0.41*
		Streptomyces	0.36*	0.33*
		Subgroup_2	-0.06	-0.05
		UTCFX1	0.29*	0.29*
Total Zn	Fungi	*Botryotrichum*	0.18*	0.17*
		Cephaliophora	0.19*	0.20*
		Saitozyma	-0.18*	-0.20*
		Trichoderma	-0.21*	-0.28*

	Bacteria	*FCPS473*	0.16*	0.20*
		RB41	-0.21*	-0.25*
		Rubrobacter	-0.17*	-0.22*
		WPS-2	0.09	0.14
Total Mo	Fungi	*Chrysosporium*	0.26*	0.24*
		Fusarium	-0.17*	-0.16*
		Pyrenochaeta	-0.18*	-0.15*
		Saitozyma	0.07	0.16*
	Bacteria	*Bradyrhizobium*	-0.16*	-0.26*
		Flavisolibacter	0.02	0.08
		Phenylobacterium	-0.18*	-0.24*
		UTCFX1	0.15*	0.27*
Total Ni	Fungi	*Acremonium*	-0.01	0.07
		Cephaliophora	0.36*	0.20*
		Saitozyma	0.02	-0.02
		Trichoderma	-0.05	-0.13

Table S3. The correlation coefficients and p-values (two-sided) of the correlations between micronutrient concentrations and the relative/absolute abundances of functional genes that are shown in the C, N, P and S pathways in Fig. 3.

Micronutrient	Gene	Absolute abundance		Relative abundance	
		Coefficient (r)	p-value	Coefficient (r)	p-value
Available Fe	*amyX*	0.20	0.009	0.21	0.006
	cdh	0.36	0	0.36	0
	narG	0.26	0	0.19	0.014
	nirS	0.25	0	0.27	0
	nifH	0.31	0	0.24	0.002
	gdhA	0.29	0	0.18	0.022
	dsrA	0.37	0	0.45	0
	dsrB	0.36	0	0.41	0
Total Mn	*manB*	0.16	0.036	0.18	0.017
	mpn	0.18	0.020	0.15	0.049
	pox	0.16	0.038	0.17	0.028
Available Cu	*cdh*	0.27	0	0.15	0.049
	narG	0.25	0	0.19	0.014
	nirK	0.19	0.014	0.23	0.002
	nirS	0.27	0	0.26	0.001
	dsrA	0.27	0	0.23	0.003
	dsrB	0.27	0	0.22	0.004
Available Zn	*napA*	0.35	0	0.21	0.005
	nirK	0.26	0.001	0.23	0.003
	bpp	0.28	0	0.17	0.031
Total Zn	*nirK*	0.17	0.026	0.23	0.001
	amoA	0.16	0.041	0.17	0.024
Total Mo	*exo-chi*	0.25	0.001	0.20	0.010
Available Mo	*narG*	0.20	0.011	0.19	0.012
	nirK	0.17	0.023	0.23	0.002
APC1	*narG*	0.28	0	0.20	0.011
	nirK	0.25	0	0.23	0.002
	nirS	0.24	0.002	0.19	0.015
	dsrA	0.30	0	0.23	0.002
	dsrB	0.30	0	0.24	0.001

Table S4. Microorganisms containing the genes involved in C, N, P and S cycling at genus level. The genera with the top 5 highest abundances across the samples and the genera identified by Deseq2 (marked with "*") are presented.

Gene		Genus	Phylum
amyX	pullulanase	*Microlunatus*	*Actinobacteria*
		Arthrobacter	*Actinobacteria*
		Pseudarthrobacter	*Actinobacteria*
		Asanoa	*Actinobacteria*
		Opitutus	*Verrucomicrobia*
		*Ralstonia**	*Proteobacteria*
manB	Mannanase	*Nocardioides**	*Actinobacteria*
		Gaiella	*Actinobacteria*
		Sphingomonas	*Proteobacteria*
		Conexibacter	*Actinobacteria*
		*Saccharopolyspora**	*Actinobacteria*
		*Aspergillus**	*Ascomycota*
		*Rubrobacter**	*Actinobacteria*
		*Actinomadura**	*Actinobacteria*
		*Streptomyces**	*Actinobacteria*
		*Ralstonia**	*Proteobacteria*
		*Anaerolinea**	*Chloroflexi*
		*Promicromonospora**	*Actinobacteria*
		*Flavisolibacter**	*Bacteroidetes*
		*Phenylobacterium**	*Proteobacteria*
cdh	Cellobiose dehydrogenase	*Roseicella*	*Proteobacteria*
		Edaphobacter	*Acidobacteria*
		Mycobacterium	*Actinobacteria*
		Rhodopila	*Proteobacteria*
		Microbispora	*Actinobacteria*
		*Streptomyces**	*Actinobacteria*
chiA	endochitinase	*Sorangium*	*Proteobacteria*
		Herpetosiphon	*Chloroflexi*
		Labilithrix	*Proteobacteria*
		Agromyces	*Actinobacteria*
		Cellvibrio	*Proteobacteria*
		*Fusarium**	*Ascomycota*
pox	Phenol oxidase	*Marmoricola*	*Actinobacteria*
		*Streptomyces**	*Actinobacteria*

		Rhodococcus	*Actinobacteria*
		Nitrospira	*Nitrospirae*
		Mycolicibacterium	*Actinobacteria*
		*Bradyrhizobium**	*Proteobacteria*
napA	periplasmic nitrate reductase	*Streptomyces**	*Actinobacteria*
		Micromonospora	*Actinobacteria*
		Kribbella	*Actinobacteria*
		Sorangium	*Proteobacteria*
		Cupriavidus	*Proteobacteria*
		*Rubrobacter**	*Actinobacteria*
		*Nocardioides**	*Actinobacteria*
narG	nitrate reductase alpha chain	*Solirubrobacter*	*Actinobacteria*
		Gaiella	*Actinobacteria*
		*Nocardioides**	*Actinobacteria*
		Patulibacter	*Actinobacteria*
		Streptomycetaceae	*Actinobacteria*
		*Bradyrhizobium**	*Proteobacteria*
		*Rubrobacter**	*Actinobacteria*
		*Actinomadura**	*Actinobacteria*
		*Saccharopolyspora**	*Actinobacteria*
		*Streptomyces**	*Actinobacteria*
		*Ralstonia**	*Proteobacteria*
		*Anaerolinea**	*Chloroflexi*
		*Promicromonospora**	*Actinobacteria*
		*Phenylobacterium**	*Proteobacteria*
nirK	nitrite reductase (NO-forming)	*Solirubrobacter*	*Actinobacteria*
		Gemmatirosa	*Gemmatimonadetes*
		*Bradyrhizobium**	*Proteobacteria*
		Gemmatimonas	*Gemmatimonadetes*
		Frankia	*Actinobacteria*
		*Rubrobacter**	*Actinobacteria*
		*Actinomadura**	*Actinobacteria*
		*Saccharopolyspora **	*Actinobacteria*
		*Streptomyces**	*Actinobacteria*
		*Nocardioides**	*Actinobacteria*
		*Ralstonia**	*Proteobacteria*
		*Anaerolinea**	*Chloroflexi*

nirS	nitrite reductase (NO-forming)	*Acidovorax*	*Proteobacteria*
		Azohydromonas	*Proteobacteria*
		Pseudomonas	*Proteobacteria*
		Ideonella	*Proteobacteria*
		Sulfuricaulis	*Proteobacteria*
		*Anaerolinea**	*Chloroflexi*
amoA	ammonia monooxygenase α-subunit	*Candidatus Nitrosocosmicus*	*Thaumarchaeota*
nifH	nitrogenase iron protein	*Anaeromyxobacter*	*Proteobacteria*
		*Bradyrhizobium**	*Proteobacteria*
		Geobacter	*Proteobacteria*
		Skermanella	*Proteobacteria*
		Sphingomonas	*Proteobacteria*
gdhA	glutamate dehydrogenase	*Gaiella*	*Actinobacteria*
		Conexibacter	*Actinobacteria*
		Acidobacterium	*Acidobacteria*
		Frankia	*Actinobacteria*
		Sandaracinus	*Proteobacteria*
		*Bradyrhizobium**	*Proteobacteria*
		*Actinomadura**	*Actinobacteria*
		*Saccharopolyspora**	*Actinobacteria*
		*Streptomyces**	*Actinobacteria*
		*Nocardioides**	*Actinobacteria*
		*Anaerolinea**	*Chloroflexi*
		*Promicromonospora**	*Actinobacteria*
		*Flavisolibacter**	*Bacteroidetes*
		*Phenylobacterium**	*Proteobacteria*
bpp	β-propeller phytase	*Solirubrobacter*	*Actinobacteria*
		Steroidobacter	*Proteobacteria*
		*Streptomyces**	*Actinobacteria*
		Lysobacter	*Proteobacteria*
		Limnoraphis	*Cyanobacteria*
		*Actinomadura**	*Actinobacteria*
		*Aspergillus**	*Ascomycota*
		*Saccharopolyspora**	*Actinobacteria*

		*Nocardioides**	*Actinobacteria*
		*Ralstonia**	*Proteobacteria*
		*Anaerolinea**	*Chloroflexi*
		*Promicromonospora**	*Actinobacteria*
		*Phenylobacterium**	*Proteobacteria*
dsrA	sulfite reductase alpha subunit	*Anaeromyxobacter*	*Proteobacteria*
		Sulfurifustis	*Proteobacteria*
		Sulfuricaulis	*Proteobacteria*
		Piscinibacter	*Proteobacteria*
		Curvibacter	*Proteobacteria*
		*Anaerolinea**	*Chloroflexi*
dsrB	sulfite reductase beta subunit	*Anaeromyxobacter*	*Proteobacteria*
		Rubrivivax	*Proteobacteria*
		Piscinibacter	*Proteobacteria*
		Ideonella	*Proteobacteria*
		Sulfuricaulis	*Proteobacteria*
		*Anaerolinea**	*Chloroflexi*

Table S5. The name and annotation of genes involved in C, N, P and S cycling measured by high-throughput qPCR based chip.

Gene name	Classification	Gene category	Function
*abf*A	C degradation	Hemicellulose	α-L-arabinofuranosidase
*amy*A	C degradation	Starch	α-amylase
*amy*X	C degradation	Starch	pullulanase
apu	C degradation	Starch	amylopullulanase
cdh	C degradation	Cellulose	cellobiose dehydrogenase
cex	C degradation	Cellulose	exoglucanase
*chi*A	C degradation	Chitin	endochitinase
exo-chi	C degradation	Chitin	exochitinase
glx	C degradation	Lignin	glyoxal oxidase
iso-plu	C degradation	Starch	Isopullulanase
lig	C degradation	Lignin	lignin peroxidase
*man*B	C degradation	Hemicellulose	β- mannanase
mpn	C degradation	Lignin	manganese peroxidase
naglu	C degradation	Cellulose	α-N-acetylglucosaminidase
pgu	C degradation	Pectin	pectinase/polygalacturonase
pox	C degradation	Lignin	phenol oxidase
sga	C degradation	Starch	glucoamylase
*xyl*A	C degradation	Hemicellulose	xylose isomerase
*acc*A	C fixation		acetyl-CoA carboxylase carboxyltransferase α subunit
*acl*B	C fixation	C fixation	ATP-citrate lyase β subunit
*acs*A	C fixation	C fixation	acetyl-coenzyme A synthetase
*acs*B	C fixation	C fixation	acetyl-CoA synthase complex β subunit
*acs*E	C fixation	C fixation	5-methyltetrahydrofolate corrinoid methyltransferase
*cda*R	C fixation	C fixation	carbohydrate diacid regulon transcriptional regulator
*frd*A	C fixation	C fixation	fumarate reductase flavoprotein subunit
*kor*A	C fixation	C fixation	2-oxoglutarate ferredoxin oxidoreductase α subunit
mct	C fixation	C fixation	mesaconyl-CoA C1-C4 CoA transferase
*mcr*A	C fixation	C fixation	methyl-coenzyme M reductase α subunit
*pcc*A	C fixation	C fixation	acetyl/propionyl-Co carboxylase alpha

*rbc*L	C fixation	C fixation	ribulose-bisphosphate carboxylase large chain
*smt*A	C fixation	C fixation	succinyl-CoA:(S)- malate CoA transferase
*mmo*X	Methane metabolism	Methane oxidation	methane monooxygenase component A alpha chain
*mxa*F	Methane metabolism	Methane production	methanol dehydrogenase (cytochrome c) subunit 1
pqq-mdh	Methane metabolism	Methane production	methanol/ethanol family PQQ-dependent dehydrogenase
*pmo*A	Methane metabolism	Methane oxidation	methane/ammonia monooxygenase subunit A
*amo*A1	N Cycling	Nitrification	ammonia monooxygenase α subunit
*amo*A2	N Cycling	Nitrification	ammonia monooxygenase α subunit
*amo*B	N Cycling	Nitrification	ammonia monooxygenase β subunit
*gdh*A	N Cycling	Organic N mineralization	glutamate dehydrogenase
hao	N Cycling	Nitrification	hydroxylamine oxidoreductase
hzo	N Cycling	Anaerobic ammonium oxidation	hydrazine oxidase
*hzs*A	N Cycling	Anaerobic ammonium oxidation	hydrazine synthase α subunit
*hzs*B	N Cycling	Anaerobic ammonium oxidation	hydrazine synthase β subunit
napA	N Cycling	Dissimilatory N reduction	periplasmic nitrate reductase
*nar*G	N Cycling	Denitrification	nitrate reductase α chain
*nas*A	N Cycling	Assimilatory N reduction	assimilatory nitrate reductase catalytic subunit
*nif*H	N Cycling	N fixation	nitrogenase iron protein
*nir*K1	N Cycling	Denitrification	nitrite reductase (NO-forming)
*nir*K2	N Cycling	Denitrification	nitrite reductase (NO-forming)
*nir*K3	N Cycling	Denitrification	nitrite reductase (NO-forming)
*nir*S1	N Cycling	Denitrification	nitrite reductase (NO-forming)
*nir*S2	N Cycling	Denitrification	nitrite reductase (NO-forming)
*nir*S3	N Cycling	Denitrification	nitrite reductase (NO-forming)
*nos*Z1	N Cycling	Denitrification	nitrous-oxide reductase
*nos*Z2	N Cycling	Denitrification	nitrous-oxide reductase
*nxr*A	N Cycling	Nitrification	nitrite oxidoreductase α subunit
*ure*C	N Cycling	Ammonification	urease
bpp	P Cycling	Organic P mineralization	β-propeller phytase

cphy	P Cycling	Organic P mineralization	ruminal cysteine phytase
gcd	P Cycling	Inorganic P solubilization	quinoprotein glucose dehydrogenase
*phn*K	P Cycling	Organic P mineralization	phosphonate transport system ATP-binding protein
*pho*D	P Cycling	Organic P mineralization	alkaline phosphatase D
*pho*X	P Cycling	Organic P mineralization	alkaline phosphatase/Pho regulon
ppk	P Cycling	Inorganic P biosynthesis	polyphosphate kinase
ppx	P Cycling	Inorganic P hydrolysis	exopolyphosphatase
*pqq*C	P Cycling	Inorganic P solubilization	pyrroloquinoline-quinone synthase
*aps*A	S Cycling	S reduction	adenosine-5’-phosphosulfate reductase α subunit
*dsr*A	S Cycling	S reduction	sulfite reductase α subunit
*dsr*B	S Cycling	S reduction	sulfite reductase β subunit
*sox*Y	S Cycling	S oxidation	sulfur-oxidizing protein
*yed*Z	S Cycling	S oxidation	sulfite oxidase

Table S6. Pearson correlation between soil total micronutrients, available micronutrients and soil macronutrients. The “*” represents that the correlation is significant at $p < 0.05$ (two-sided). The number in the table represents the correlation coefficient (r).

	TFe	TMn	TCu	TZn	TMo
TMn	0.26*				
TCu	0.72*	0.27*			
TZn	0.41*	0.47*	0.53*		
TMo	0.50*	-0.08	0.36*	0.31*	
TNi	0.57*	0.39*	0.77*	0.28*	0.17

	AFe	AMn	ACu	AZn	AMo
AMn	0.30*				
ACu	0.48*	0.04			
AZn	0.33*	0.05	0.55*		
AMo	0.27*	-0.04	0.46*	0.57*	
ANi	0.44*	0.43*	0.31*	0.10	-0.01

	TP	TN	TC	C:N	C:P
TN	0.20*				
TC	0.26*	0.56*			
C:N	-0.03	-0.31*	0.08		
C:P	-0.40*	0.19*	0.38*	-0.02	
N:P	-0.35*	0.59*	0.24*	-0.27*	0.80*

Table S7. The differences in the overall microbial community revealed by PCoA between the soils with low, medium and high concentrations of available micronutrients. The low and high groups included the 60 samples with relatively lowest and highest micronutrient concentrations among 180 samples respectively. The other 60 samples were included in medium groups.

Micronutrient	Kingdom	Groups	p-value (two-sided)
Available Fe	Bacteria	Low vs High	0.026
		Medium vs High	0.700
		Low vs Medium	0.010
	Fungi	Low vs High	0.642
		Medium vs High	0.078
		Low vs Medium	0.012
Available Mn	Bacteria	Low vs High	0.450
		Medium vs High	0.898
		Low vs Medium	0.517
	Fungi	Low vs High	0.475
		Medium vs High	0.162
		Low vs Medium	0.034
Available Cu	Bacteria	Low vs High	0
		Medium vs High	0
		Low vs Medium	0.142
	Fungi	Low vs High	0.049
		Medium vs High	0.035
		Low vs Medium	0.720
Available Zn	Bacteria	Low vs High	0.125
		Medium vs High	0.083
		Low vs Medium	0.003
	Fungi	Low vs High	0.001
		Medium vs High	0.001
		Low vs Medium	0.817
Available Mo	Bacteria	Low vs High	0.022
		Medium vs High	0.614
		Low vs Medium	0.092
	Fungi	Low vs High	0.013
		Medium vs High	0.886
		Low vs Medium	0.021
Available Ni	Bacteria	Low vs High	0.009
		Medium vs High	0.080
		Low vs Medium	0.335
	Fungi	Low vs High	0.045
		Medium vs High	0.453
		Low vs Medium	0.205

Table S8. The differences in the overall microbial community revealed by PCoA between the soils with low, medium and high concentrations of total micronutrients. The low and high groups included the 60 samples with relatively lowest and highest micronutrient concentrations among 180 samples respectively. The other 60 samples were included in medium groups.

Micronutrient	Kingdom	Groups	p value (two-sided)
Total Fe	Bacteria	Low vs High	0.660
		Medium vs High	0.535
		Low vs Medium	0.287
	Fungi	Low vs High	0.002
		Medium vs High	0.006
		Low vs Medium	0.682
Total Mn	Bacteria	Low vs High	0.159
		Medium vs High	0.214
		Low vs Medium	0.006
	Fungi	Low vs High	0.003
		Medium vs High	0.070
		Low vs Medium	0.170
Total Cu	Bacteria	Low vs High	0.046
		Medium vs High	0.058
		Low vs Medium	0.930
	Fungi	Low vs High	0.002
		Medium vs High	0.047
		Low vs Medium	0.224
Total Zn	Bacteria	Low vs High	0.001
		Medium vs High	0.433
		Low vs Medium	0.011
	Fungi	Low vs High	0.028
		Medium vs High	0.002
		Low vs Medium	0.231
Total Mo	Bacteria	Low vs High	0.041
		Medium vs High	0.109
		Low vs Medium	0.002
	Fungi	Low vs High	0.008
		Medium vs High	0.004
		Low vs Medium	0.669
Total Ni	Bacteria	Low vs High	0.037
		Medium vs High	0.363
		Low vs Medium	0.208
	Fungi	Low vs High	0
		Medium vs High	0.093
		Low vs Medium	0.029
