# Supplementary material for: Metallic micronutrients are associated with the structure and function of the soil microbiome
Source: Nat Commun. 2023 Dec 20;14:8456. doi: 10.1038/s41467-023-44182-2 (PMC10730613; doi:10.1038/s41467-023-44182-2)
Supplement: Supplementary file 3 — Reporting Summary [file 41467_2023_44182_MOESM3_ESM.pdf]

nature portfolio

Corresponding author(s): Jianming Xu

Last updated by author(s): Nov 21, 2023

Reporting Summary

Nature Portfolio wishes to improve the reproducibility of the work that we publish. This form provides structure for consistency and transparency in reporting. For further information on Nature Portfolio policies, see our Editorial Policies and the Editorial Policy Checklist.

Statistics

For all statistical analyses, confirm that the following items are present in the figure legend, table legend, main text, or Methods section.

n/a	Confirmed	
☐	☒	The exact sample size (*n*) for each experimental group/condition, given as a discrete number and unit of measurement
☐	☒	A statement on whether measurements were taken from distinct samples or whether the same sample was measured repeatedly
☐	☒	The statistical test(s) used AND whether they are one- or two-sided<br>*Only common tests should be described solely by name; describe more complex techniques in the Methods section.*
☐	☒	A description of all covariates tested
☐	☒	A description of any assumptions or corrections, such as tests of normality and adjustment for multiple comparisons
☐	☒	A full description of the statistical parameters including central tendency (e.g. means) or other basic estimates (e.g. regression coefficient) AND variation (e.g. standard deviation) or associated estimates of uncertainty (e.g. confidence intervals)
☐	☒	For null hypothesis testing, the test statistic (e.g. *F*, *t*, *r*) with confidence intervals, effect sizes, degrees of freedom and *P* value noted<br>*Give P values as exact values whenever suitable.*
☒	☐	For Bayesian analysis, information on the choice of priors and Markov chain Monte Carlo settings
☒	☐	For hierarchical and complex designs, identification of the appropriate level for tests and full reporting of outcomes
☐	☒	Estimates of effect sizes (e.g. Cohen's *d*, Pearson's *r*), indicating how they were calculated

Our web collection on statistics for biologists contains articles on many of the points above.

Software and code

Policy information about availability of computer code

Data collection	No software was used to collect the data in this study.
Data analysis	Amplicon sequencing data processing was done using QIIME 2 pipeline (2022.11). Metagnomic sequencing data processing was done using Megahit (version 1.1.2), Prodigal (version 2.6.3) and Diamond (version 0.8.35). Network construction and comparison were done using R 4.1.0. Structural equation modeling was constructed using SPSS AMOS 24.0 Statistical analyses were done using R 4.1.0 and SPSS (version 24.0).The codes for sequencing analyses are publicly available in the Figshare database at https://doi.org/10.6084/m9.figshare.24596892.v1.

For manuscripts utilizing custom algorithms or software that are central to the research but not yet described in published literature, software must be made available to editors and reviewers. We strongly encourage code deposition in a community repository (e.g. GitHub). See the Nature Portfolio guidelines for submitting code & software for further information.

Data

Policy information about availability of data

All manuscripts must include a data availability statement. This statement should provide the following information, where applicable:

- Accession codes, unique identifiers, or web links for publicly available datasets
- A description of any restrictions on data availability
- For clinical datasets or third party data, please ensure that the statement adheres to our policy

Raw sequencing data were deposited in the Sequence Read Archive (SRA) with the accession number: PRJNA924284 (16S), PRJNA940307 (ITS) and PRJNA1035420 (metagenomics).The source data used in this study are available in the Figshare database at https://doi.org/10.6084/m9.figshare.24597126.v1. The database of silva

used for taxonomy assignments are available online (https://www.arb-silva.de). The database of Kyoto Encyclopedia of Genes and Genomes (KEGG) for functional assignment is available online (https://www.genome.jp/kegg/).

Research involving human participants, their data, or biological material

Policy information about studies with human participants or human data. See also policy information about sex, gender (identity/presentation), and sexual orientation and race, ethnicity and racism.

Reporting on sex and gender	N/A
Reporting on race, ethnicity, or other socially relevant groupings	N/A
Population characteristics	N/A
Recruitment	N/A
Ethics oversight	N/A

Note that full information on the approval of the study protocol must also be provided in the manuscript.

Field-specific reporting

Please select the one below that is the best fit for your research. If you are not sure, read the appropriate sections before making your selection.

☐ Life sciences ☐ Behavioural & social sciences ☒ Ecological, evolutionary & environmental sciences

For a reference copy of the document with all sections, see nature.com/documents/nr-reporting-summary-flat.pdf

Ecological, evolutionary & environmental sciences study design

All studies must disclose on these points even when the disclosure is negative.

Study description	Surface soils were collected from 180 sites across China, with a span of 32.5o longitude and 33.4o latitude. These locations vary with different climatic conditions (from cold to tropical zones), soil types (from highly weathering to less weathering) and vegetation covers (crops, grass, and forests). The mean annual precipitation and temperature ranges from 395 to 2486 mm and -2.7 to 27.9 ℃, respectively.
Research sample	Soil samples were collected using a standard and uniform sampling method.<br>Soil microbial DNA was extracted from each sample in order to investigate microbial community composition and functional genes.
Sampling strategy	Surface soil samples (0-20cm) , bulk soils, were taken using a standard soil corer and soil cores were pooled to form a composite sample to decrease spatial heterogeneity.
Data collection	Soil total concentration of metallic micronutrients including Fe, Mn, Cu, Zn, Mo and Ni were determined according to de Santiago-Martín et al. 2015. Soil DNA samples was sent for 16S, ITS and metagenomic sequencing on the Illumina paired-end platform with the instrument of NovaSeq 6000. The absolute abundance of genes involved in C, N, P and S cycling were measured using a high-throughput qPCR based chip (QMEC) on SmartChip Real-Time PCR System. Other soil property measurements were described in the manuscript.
Timing and spatial scale	Surface soils were collected from 180 sites across China. Some soil samples were collected in 2018, and some samples were collected during 2019-2020. These samples were well-distributed across China.
Data exclusions	No data were excluded.
Reproducibility	The measurements of soil parameters are described in the methods. All files and code required to repeat the experiments are provided in Figshare.
Randomization	This study investigates the relationship between soil metallic micronutrients with the structure and function of the soil microbiome. The soil samples were uniformly-distributed across China.
Blinding	Blinding is not applicable this study, as the results in our study are quantitative and do not need subjective judgment and interpretation. We also blinded samples during measurements by putting a code so nobody knew what the exact samples were.

Did the study involve field work? ☒ Yes ☐ No

Field work, collection and transport

Field conditions	Soils were collected from 180 sites across China, with a span of 32.5o longitude and 33.4o latitude. These locations vary with different climatic conditions (from cold to tropical zones), soil types (from highly weathering to less weathering) and vegetation covers (crops, grass, and forests). The mean annual precipitation and temperature ranges from 395 to 2486 mm and -2.7 to 27.9℃, respectively.
Location	Details regarding location of the sampling sites are provided in Figshare database at https://doi.org/10.6084/m9.figshare.24597126.v1
Access & import/export	Open areas were accessed by sampling team, and therefore no permits were required. The soils samples were immediately transported to the laboratory on ice.
Disturbance	No disturbance was caused in this study

Reporting for specific materials, systems and methods

We require information from authors about some types of materials, experimental systems and methods used in many studies. Here, indicate whether each material, system or method listed is relevant to your study. If you are not sure if a list item applies to your research, read the appropriate section before selecting a response.

Materials & experimental systems

n/a	Involved in the study
☒	☐ Antibodies
☒	☐ Eukaryotic cell lines
☒	☐ Palaeontology and archaeology
☒	☐ Animals and other organisms
☒	☐ Clinical data
☒	☐ Dual use research of concern
☒	☐ Plants

Methods

n/a	Involved in the study
☒	☐ ChIP-seq
☒	☐ Flow cytometry
☒	☐ MRI-based neuroimaging
